# *Conus coronatus* and *Conus frigidus* Venom: A New Source of Conopeptides with Analgesic Activity

**Published:** 2020

**Authors:** Halimeh Rajabi, Hossein Zolgharnein, Mohammad Taghi Ronagh, Jamshid Amiri Moghaddam, Max Crüsemann

**Affiliations:** 1. Abadan University of Medical Sciences, Abadan, Iran; 2. Khorramshahr University of Marine Science and Technology, Khorramshahr, Iran; 3. Leibniz Institute for Natural Product Research and Infection Biology- Hans Knöll Institute, Jena, Germany; 4. Institute for Pharmaceutical Biology, University of Bonn, Bonn, Germany

**Keywords:** Analgesics, Conotoxin, *Conus frigidus*, Pain, Venoms

## Abstract

**Background:**

Cone snails are a natural source of complex peptides with analgesic properties called conotoxins. These peptides are secreted in a complex venomic mixture and are predominantly smaller than 5 *kDa*. The present study aimed to document the analgesic activity of two species of *Conus coronatus* (*C. coronatus*) and *Conus frigidus* (*C. frigidus*) venom collected off the Iranian coast in a mouse behavioral test.

**Methods:**

Conotoxin containing fractions was extracted from the venom ducts and initially purified by column chromatography. The analgesic effect of the fractions was determined on formalin pain model and hot-plate test.

**Results:**

The results led to the identification of four fractions with analgesic activity in *C. coronatus* and two in *C. frigidus*. Only one fraction was able to reduce the flinching and licking in both acute pain and chronic pain phases of the formalin test. Moreover, the activity of this fraction remained 30 minutes on the hot-plate test. Purification of the fractions was carried out by RP-HPLC. LC-ESI-MS analysis of the fractions showed that the conotoxins of the analgesic fraction had molecular weights not previously reported.

**Conclusion:**

The findings give insight into the venom of two previously under-investigated *Conus* species and reveal the therapeutic potential of the containing conopeptides.

## Introduction

Cone snails are a group of marine invertebrates with about 803 poisonous species ^1,2^ which produce highly toxic venoms for defense and preying. These venoms are mixtures of bioactive peptide compounds with molecular weights of less than 5 *kDa* ^3–6^ secreted by cells of the venom apparatus which is an appendage of the snail’s digestive system ^7–9^. In addition, other tissues such as salivary glands contain poisonous compounds that contribute to the venom content ^10^. Cone snails often inject small amounts of venoms (about 5 *μl*), which usually consist of a complex combination of conotoxins as a defensive mechanism against hunters. The conotoxins are interesting candidates as drug leads ^11,12^ involved in analgesic, antiepileptic, epilepsy, cardio and neuroprotective activity and myocardial infarction ^4,13–16^. Conotoxins are structurally diverse and classified according to three schemes: the ER signal sequence similarities of the conotoxin precursors (gene superfamilies), the cysteine patterns of mature conotoxins and their disulfide connections (cysteine frameworks), and finally the specificities to pharmacological targets (pharmacological families).

Currently, 28 super-gene families are described. The pharmacological targets of some conotoxins have been identified ^14,17^. Although several hundred conotoxins are reported from *Conus* species, few of them have been characterized for their potential pain-relieving roles. This potential is related to the high potency of conotoxins to specifically block individual types of excitable channels on nerve cells, some of which give rise to pain states ^14^. Some of the analgesic conotoxins studied to date are: i) α-conotoxins; ImI and ImII from *Conus imperialis* (*C. imperialis*) ^18^, ii) μ-conotoxins; CnIIIC from *Conus consors* (*C. consors*) ^4^, GIIIA from *Conus geographus* (*C. geographus*) and PIIIA from *Conus purpurascens* (*C. purpurascens*) ^19^, iii) ω-conotoxin; SVIB from *Conus striatus* (*C. striatus*), GVIA from *Conus geographus* (*C. geographus*) and MVIIA from *Conus magus* (*C. magus*). The pain killer “Ziconotide”, derived from ω-conotoxin MVIIA, was approved in December 2004 by the U.S. Food and Drug Administration ^20^. Due to various pharmacological activities and the large structural diversity, conotoxins represent a valuable source for the discovery of novel drug leads ^10,21,22^. Here, an attempt was made to explore the therapeutic potential of cone snail venom from the Iranian coast, which remains a largely uninvestigated source for these toxins.

**Figure 1.**
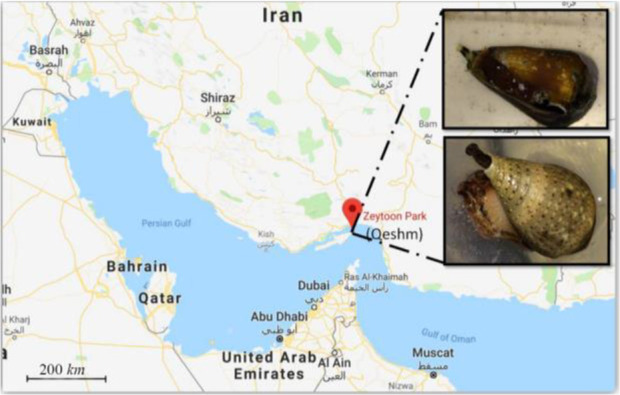
Sampling area for *C. coronatus* (bottom picture) and *C. frigidus* (upper picture) used in this study.

*C. coronatus* and *C. frigidus* are dominant species on Qeshm Island, in the northern part of the Persian Gulf (Figure 1) ^23^ but to our knowledge, there is no study about analgesic activity of these species. Thus, our goal was to collect the venom of these cone snail species and investigate their chemical and pharmacological properties.

## Materials and Methods

### Sample collection

Thirty-nine specimens of *C. coronatus* and twenty-five specimens of *C. frigidus* with total length of about 2.4±0.2 and 5.4±1.2 *cm*, respectively were collected from coastal waters of Zeyton Park in Qeshm Island (Location: N 26055.631; E 056015.209, south of Iran) in September 2016. The specimens were kept alive in salt water and transferred to the biotechnology laboratory of Hormozgan University, Iran. Then, dissection and isolation of the venom duct was performed on sterile conditions. Venom ducts of each species were lyophilized separately and transferred on ice to the bio-technology laboratory of Khorramshahr University of Marine Science and Technology, Iran for further procedures.

### Conotoxin extraction

Conotoxin extraction was performed by addition of deoxygenated cold aqueous acetonitrile solution (40%) to the venom duct and homogenization (Homogenizer Silent Crusher, Heidolph, Germany) at 16,000×*rpm* for 5 *min*. Then, the mixture was centrifuged at 10,000×*g* for 20 *min* and the supernatant containing the conotoxins was lyophilized using freeze dryer (Model Christ, 2 alpha, Germany) for 24 *hr* at −56°*C* ^9^. The protein concentration was determined by using the Bovine Serum Albumin (BSA) protein quantification method according to Bradford ^24^.

### Purification

#### Gel filtration:

The first separation of the conotoxins was carried out using open gel filtration column chromatography. In order to prepare the column (2.5×90 *cm*), 10 *g* of Sephadex G-25 resin was suspended in 50 *mM* Tris buffer (pH=8.5). The column was equilibrated with 50 *mM* Tris buffer with pH=8.5. The flowthrough speed was set to 0.3 *ml per min*. After loading the lyophilized protein to the column, 2 *ml* of fractions were collected for 10 *hr* and then lyophilized again ^25^.

#### RP-HPLC purification:

Reverse-phase HPLC [Pump model; K-100, UV detector model 2550 and manual injector (Knauer, Germany)] was used for further purification of conotoxins. Next, 100 *μl* of the active fraction C2 was injected to a flow rate of 1 *ml/min* on an analytical C_18_ column (Particle size: 5 *μ*, pore size: 300 Å, column size: 4.6×250 *mm*) (Coulter, USA). HPLC begins with 90% H_2_O containing 0.05% TFA with a gradient to 100% acetonitrile containing 0.05% TFA over 90 *min*. The data was analyzed using ChromGate v 3.3.2 ^9^.

### LC-ESI-MS analysis

Samples were analyzed by LC-ESI-MS on a micrOTOF-Q III mass spectrometer (Bruker) with ESI-source coupled with a HPLC Dionex UltiMate 3000 (Thermo Scientific) using a EC100/2 Nucleoshell RP18 Gravity 2.7 *μm* (Macherey-Nagel, Düren, Germany). MS data were acquired over a range from 100–3000 *m/z* in positive mode. The column temperature was 25°*C* and the LC conditions used were as follows; eluent A was water with 0.1% acetic acid, while eluent B was acetonitrile containing 0.1% acetic acid. A gradient of 0–60% of eluent B in 12 *min* with a flow rate of 0.3 *ml/min* was used. Analysis of the MS data was performed by Bruker Compass Data Analysis 4.1. Monoisotopic masses were compared with similar monoisotopic masses of the peptide sequences/structures which are recorded in ConoServer database (http://www.conoserver.org/index.php?page=stats&tab=organisms). Additionally, detected masses were explored for possible fingerprints using “Search by peptide mass” located in the ConoServer database (http://www.cono-server.org/index.php?page=fingerprint) ^17^.

### Analgesic test

Male mice in the weight range of 20–25 *g* were provided from Ahvaz University of Medical Sciences, and used further according to the guidelines for the care and maintenance of laboratory animals. Mice were kept in the animal house under specified conditions (light cycle of 12 *hr* darkness and 12 *hr* light, temperature of 23±2°*C* and humidity of 40–50%) and were fed by a special animal pellet.

#### Formalin test:

Lyophilized crude extract and purified fractions were dissolved in deionized water. Intraperitoneal (IP) injection was performed using 100 *μl* of the crude extract (1 *mg/kg* dose), purified fractions (0.5 *mg/kg* dose), normal saline (negative control) and morphine (positive control, 2.5 *mg/kg* dose) per 10 *g* mouse weight. Seven mice were used in each experimental group. Each mouse was placed in a box with dimensions of 30×30×30 *cm*^3^. After one hour, 10 *μl* of formalin 2.5% per 10 *g* mouse weight was subcutaneously injected into the right paw of the mouse. Then, the pain response was assessed for one hour. Number of flinches and licking time were considered as pain response ^13,26,27^.

#### Hot plate test:

The thermal pain response was assessed 30 *min* after the injection and at intervals (0, 15, 30, 45, and 60 *min*), then the latency was assessed, and compared with the base tolerance. Hot plate (RB200, Chengdu Taiment Techonology Inc, China) was used to test the thermal pain and the temperature was kept constantly at 55±1°*C*. Licking and jumping up were considered as response to the painful stimuli. If the reaction time is shorter, the heat pain is more severe. Seven mice were used in each experimental group. Each mouse was placed on a hot plate and the time response to the painful stimuli was measured. This amount was the baseline latency of animals. The test was performed in the following groups: normal saline (Negative control), fraction C2 (0.05 and 0.1 *mg/kg*), nicotine (0.005, 0.05 and 0.1 *mg/kg*). Cut off time was considered to be about 60 *s* ^27,28^.

### Statistical analysis

Values were expressed as mean±SEM of seven animals in each group. All the data were statistically compared with the controls by one-way ANOVA, where p<0.05 was considered to be statistically significant. Statistical analyses were performed using Graph-Pad Prism software version 6.

## Results

### Extraction and purification of conotoxins

**Figure S1.**
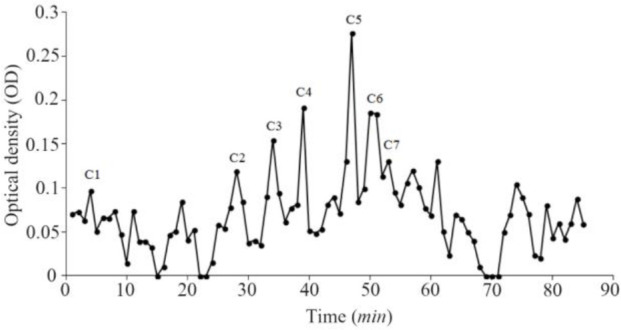
Sephadex G-25 chromatogram of *C. coronatus* extract (220 *nm*).

**Figure S2.**
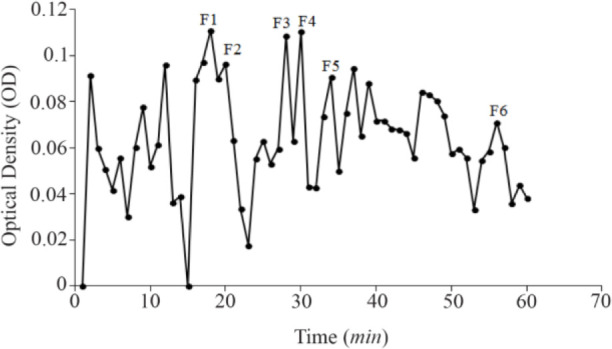
Sephadex G-25 chromatogram of *C. frigidus* extract (220 *nm*).

In total, 3.2 *mg* of crude extract from *C. coronatus* and 2.6 *mg* from *C. frigidus* was yielded from the venom ducts. The crude extracts were purified using Sephadex G-25. Next, 85 fractions for *C. coronatus* with seven main peaks (C1–C7) and 61 fractions for *C. frigidus* with five main peaks (F1–F5) were collected over 10 h and their UV absorption was spectrometrically determined (Figure S1 and S2).

**Figure S3.**
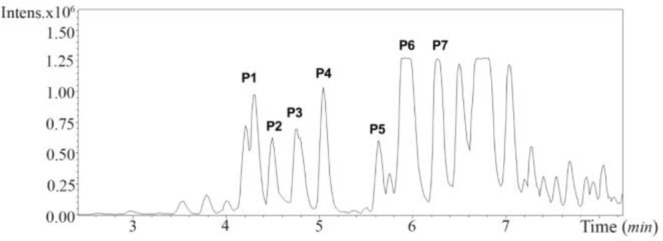
RP-HPLC chromatogram of fraction C2 from *C. coronatus*

Due to the promising analgesic results (4.2 section), fraction C2 of *C. coronatus* was further purified by RP-HPLC (Figure S3). The collected subfractions of fraction C2 were analyzed using LC-ESI-MS and high molecular weight compounds were detected. The monoisotopic masses of the putative conotoxins are presented in table 1. None of the detected monoisotopic masses had been reported in ConoServer database before.

**Table 1.** Summary of high-molecular weight peaks from *C. coronatus* C2

**Peak number**	**Rt (*min*)**	**Detected masses (*m/z*) and corresponding molecular weight below in bold (*Da*)**			
**P1**						
	4,2	739.52		851.51	
		**738.51**		**850.50**	
**P2**						
	4,4	627.53		765.54	
		**626.52**		**764.53**	
**P3**						
	4,8	903.54		979.56	1055.58
		**902.53**		**978.55**	**1054.57**
**P4**						
	5	1055.58			
		**1054.57**			
**P5**						
	5,6	907.58			
		**906.57**			
**P6**						
	5,9	907.58	1360.87 (m/2)	1587.51(m/2)	1814.16 (m/2)
		**906.57**	**2719.74**	**3173.02**	**3626.32**
		1965.25 (m/3)		2040.81 (m/2)	2116.35 (m/3)
		**5772.75**		**4079.6**	**6345.9**
**P7**						
	6,3	959,61			
		**958.60**			

### Analgesic test

**Figure 2.**
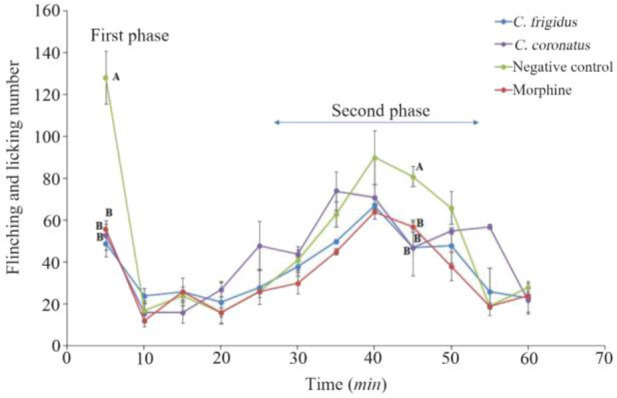
Subcutaneous injection of formalin in mice as a pain model in different mice groups; negative control (Normal saline), *Conus* extracts (1 *mg/kg*) and morphine as positive control (2.5 *mg/kg*). Different letters indicate significant differences between groups in the first and second phase of pain (p<0.05). In the second phase of pain (Chronic phase), AUC (Area under curve) was calculated.

To explore the analgesic effects of conotoxins in the crude extract, formalin test was used on mice and compared with control groups. Generally, in the first phase (acute phase) of formalin injection, the flinching and licking increased, and then gradually decreased. In the second phase (chronic phase), the flinching and licking increased again, peaking after 35–45 *min*. Finally, the flinching and licking decreased after 60 *min* (Figure 2). As shown in figure 2, in mice which were injected with *Conus* extracts and morphine, both acute (first) and chronic (second) phases of pain decreased by 2.5 and 1.5 times, respectively (p<0.05) in comparison to the negative control group which was injected with normal saline.

**Figure 3.**
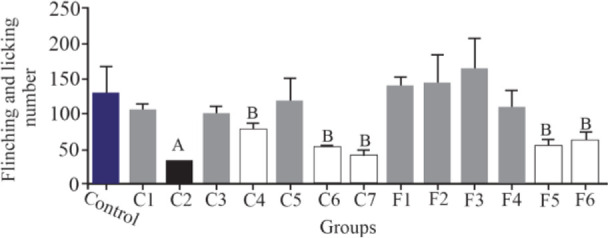
The comparison of number of flinches and licking time among different fractions of *C. coronatus* (C1–C7) and *C. frigidus* (F1–F6) with subcutaneous injection in mice with formalin in the first phase (7 mice were used for each group. Different symptoms indicate significant differences) (p<0.05).

To assess the analgesic effect of the purified fractions, a formalin test with an initial dose of 0.5 *mg/kg* was performed for seven fractions resulting from the partially purified extract (C1–C7) of *C. coronatus* and six fractions of *C. frigidus* (F1–F6). The results showed that the fractions of C2, C4, C6, C7, F5 and F6 had analgesic effects on the first phase of the formalin test. The fraction number C2 had the most analgesic effect compared to others in the first phase (Figure 3).

**Figure 4.**
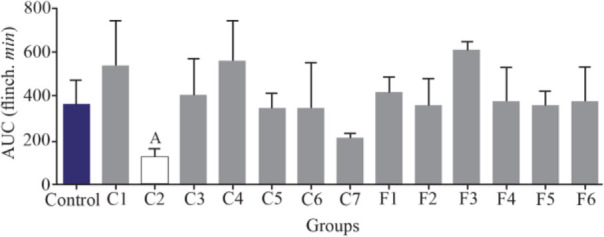
Number of flinches and licking time as AUC is calculated among different fractions for the second phase of formalin test in *C. coronatus* (C1–C7) and *C. frigidus* (F1–F6). Seven mice were used for each group, Different symptoms indicate significant differences (p<0.05).

Interestingly, only fraction C2 could decrease the second pain phase significantly (p<0.05). All the other fractions could not substantially decrease the second pain phase of the formalin test and were similar to the control group (Figure 4).

### Thermal pain test of fraction C2

**Figure 5.**
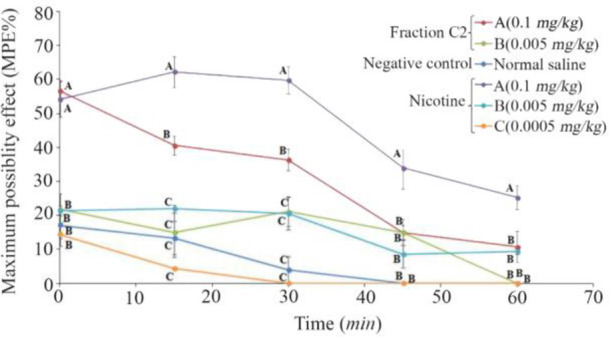
Thermal pain test of fraction C2 (*C. coronatus*) and nicotine (Positive control) with different dosages (*mg/kg*). Seven mice were used for each group and different letters indicate significant difference between groups (p<0.05).

Due to strong analgesic effects of fraction C2 in formalin test and available materials, a thermal pain test was performed on this fraction. Different doses of fraction C2 containing conopeptide (C2-A: 0.1 and C2-B: 0.005 *mg/kg*) and nicotine (N-A: 0.1, N-B: 0.005 and N-C: 0.0005 *mg/kg*) were applied. C2-A and N-A had a significant analgesic effect in the interval of 0–60 *min*. At time 0, the analgesic effect of C2-A was similar to the effect of N-A, and significantly different from the other doses. Also, at 15 and 30 *min* intervals, the analgesic effects were observed at this dose and until the end of the 30 *min* duration. The mice which were injected lower dosages of nicotine and C2 (N-B, N-C and C2-B) were similar to the negative control group that was injected with normal saline. Also, the analgesic effect of N-A remained until the end of the 60 *min* test period (Figure 5).

## Discussion

Basic *in vivo* pain research relies on animal experiments and these tests have led to many contributions to the medical progress. Due to the genetic differences in animals, their individual tolerance to pain varies, but the variations are lower than in other behavioral tests. In these experiments, electrical, thermal, mechanical and chemical stimuli are used, and the differences in pain expression are associated with nerve fibers as well as the molecular mechanisms and pharmacological and genetic manipulations ^29,30^. In relation to chemical stimulants, different compounds can be used to evaluate the analgesic effects which are measurable by using behavioral scoring ^28,31^.

The most common test using chemical stimuli is the formalin test. The benefit of the formalin test compared to other tests is the possibility of reviewing the period and duration of the pain (acute and inflammatory). Formalin primarily causes pain via the TRPA1 receptor which is a member of the TRP family of ion channels ^29,32^.

In the present study, the formalin test was used on mice as the behavioral model, and the *Conus* extracts were injected IP. It should be noted that the intracranial and spinal injection represents a more specific binding to their individual receptors ^4,15,33^. Pain response to formalin administration was biphasic, as expected (Figure 2). The mechanism of pain induced by formalin involves a set of central and peripheral factors. The first phase is a rapid response to environmental stimuli and the second phase is a result of inflammatory mediators and functional changes inducing central pain ^13,25,26,32^. Administration of both *Conus* crude extracts led to less pain induced by formalin similar to the injection of morphine, which is comparable with the results of other studies on different cone snails ^4,34–36^.

According to the pattern of pain in investigated mice, there were slight differences in pain models (in time) which are influenced by genetic factors ^37^. Pain includes various stages and certain compounds are involved, including neurotransmitters and enzymes. Some of these compounds result in increased sensitivity and others reduce sensitivity to pain. An increase or decrease in the production of these compounds is influenced by genetics ^38^. Moreover, many studies point to analgesic effects of different species of cone snails in different behavioral models. For example, in 2014, Tabaraki *et al* examined the effect of intrathecal injection of different doses of crude extract of *Conus textile* (*C. textile*) on formalin test and found that 10 *ng* of toxin had similar effects of morphine on pain relief in mice ^15^. In another study, Kumar *et al* measured the analgesic activity of *Conus lentiginosus* (*C. lentiginosus*) in a Tail Flick test and showed that the analgesic effect of the semi-purified venom of this species was three times more effective than Paracetamol ^16^. Therefore, in the current study, after analyzing the analgesic effects of the crude extract, purification of the extract was performed and the analgesic effects of the resulting fractions were also investigated.

Experiments on the analgesic activity of partially purified extracts of *C. coronatus* (C1–C7) and *C. frigidus* (F1–F6) showed that the fractions number C2, C4, C6, C7, F5 and F6 had an analgesic effect in the first phase (Figure 3) and only fraction number C2 was able to decrease the pain in the second phase, too (Figure 4). After IP injection to mice and formalin test, the μ-conotoxin KIIIA showed a significant decrease in licking and flinching of mice during both phases (similar to the fraction C2). This peptide was later found to block sodium channels ^30^.

In the hot plate test, analgesic effects of the fraction C2 remained for 30 *min* (Figure 5). It has been reported that the analgesic effects of *Conus regularis* (*C. regularis*) conotoxin that was injected IP (at dose of 0.85 *mg/kg*) was still remaining for about 15 *min*, which corresponds to an N-type calcium channel blocking conotoxin ^25^. Also, the same dose of nicotine had more analgesic effect. Compounds such as nicotine, which are agonists of the cholinergic receptor activities, have anti-inflammatory effects ^39^. High doses may cause adverse effects in behavioral models. McIntosh *et al* reported that intracranial injection of 30 *nmol* mr10a conotoxin in hot plate test causes seizures. However, injection of 2 *nmol* had analgesic effects of about 39.5±13.5 S ^40^.

ESI-MS analysis of the purified fraction C2 showed that seven peaks contained compounds with a molecular weight between 0.5 to 6.4 *kDa*. Other compounds in the fraction were probably secondary compounds that played a role in the maturation of conotoxin, or were small portions of protein precursor before maturity in the venom apparatus ^10,41^. Heghinian and Mari ^12^ reported that purified fractions of cone snail venom can contain several different peptides with different biological activities ^6,12^. Based on the molecular weights obtained from LC-HR-ESI-MS analysis, compound of fraction C2 did not show similarity to any conotoxins in ConoServer database (Table 1), so they likely represent novel conotoxin structures that will be further isolated and analyzed in future studies.

## Conclusion

The analgesic effects of *C. coronatus* and *C. frigidus* venom were observed in this study which directed us towards the goodpotential of therapeutic molecules. In future studies, more information about the conotoxin(s) with analgesic properties, their structure and function as well as their toxicity need to be obtained. Given that *C. coronatus* is one of the dominant species in the Persian Gulf, and is accessible for researchers, this is an attractive source for further research on conotoxins.

## References

[1] Pillandre N Bouchet P Duda TF Kauferstein S Kohn A Olivera BM Molecular phylogeny and evolution of the cone snails. Mol Phylogenet Evol 2014;78:290–303. 24878223 10.1016/j.ympev.2014.05.023 PMC5556946

[2] Pillandre N Duda TF Meyer C Olivera BM Bouchet P One, four or 100 genera? A new classification of the cone snails. J Molluscan Stud 2015;81(1):1–23. 26300576 10.1093/mollus/eyu055 PMC4541476

[3] Dobson R Collodor M Gilles N Turtoi A De Pauw E Quinton L Secretion and maturation of conotoxins in the venom ducts of Conus textile. Toxicon 2012;60(8):1370–1379. 23031820 10.1016/j.toxicon.2012.09.013

[4] Favreau P Benoit E Hocking HG Carlier L Hoedt DD Leipold E A novel µ-conotoxin, CnШC, exerts potent and preferential inhibition of NaV 1.2/1.4 channels and blocks neuronal nicotinic acetylcholine receptors. British J Pharm 2012;166(5):1654–1668. 10.1111/j.1476-5381.2012.01837.x PMC3419909 22229737

[5] Neves J Campos A Osorio H Antunes A Vitor V Conotoxin from Cape Verde Conus crotchii. Mar Drug 2013; 11(6):2203–2215. 10.3390/md11062203 PMC3721229 23783403

[6] Rodriguez AM Dutertre S Lewis RJ Mari F Intraspecific variation in Conus purpurascens injected venom using LC/MALDI-TOF-MS and LC-ESI-Triple TOF-MS. Anal Bioanal Chem 2015;407(20):6105–6116. 26048056 10.1007/s00216-015-8787-y

[7] Marshall J Kelley WP Rubakhin SS Bingham JP Sweedler JV Gilly WF Anatomical correlates of venom production in Conus californicus. Biol Bull 2002;203(1):27–41. 12200253 10.2307/1543455

[8] Page LR Metamorphic remodeling of a planktotrophic larva to produce the predatory feeding system of a cone snail (Mollusca, Neogastropoda). Biol Bull 2011,221(2): 176–188. 22042436 10.1086/BBLv221n2p176

[9] Tayo LL Lu B Cruz LJ Yates JR 3rd Proteomic analysis provides insights on venom processing in Conus textile. J Proteome Res 2010;9(5):2292–2301. 20334424 10.1021/pr901032r PMC2909976

[10] Biass D Violette A Hulo N Lisacek F Favreau P Stocklin R Uncovering intense protein diversification in a cone snail venom gland using an integrative venomics approach. J Proteome Res 2015;14(2):628–638. 25536169 10.1021/pr500583u

[11] Dutertre S Jin AH Vetter I Hamilton B Sunagar K Lavergne V Evolution of separate predation and defence evoked venoms in carnivorous cone snails. Nat Commun 2014;5:1–9. 10.1038/ncomms4521 PMC3973120 24662800

[12] Heghinian M Mari F Discovery and biological characterization of conotoxin from the venom of Conus brunneus in Drosophila melanogaster. Presented for the Ph.D., Florida Atlantic University 2014.

[13] Bernaldez J López O Licea A Salceda E Arellano R.O Vega R Electrophysiological characterization of a novel small peptide from the venom of Conus californicus that targets voltage-gated neuronal Ca2+ channels. Toxicon 2013;57(1):60–67. 10.1016/j.toxicon.2010.09.015 20920515

[14] Lewis RJ Dutertre S Vetter I Christie MJ Conus venom peptide pharmacology. Pharm Rev 2012;64(2):259–298. 22407615 10.1124/pr.111.005322

[15] Tabaraki N Shahbazzadeh D Moradi AM Vosughi G Mostafavi PG Analgesic effect of Persian Gulf Conus textile venom. Iran J Basic Med Sci 2014;17(10):793–797. 25729549 PMC4340988

[16] Kumar P Venkateshvaran K Srivastava PP Nayak SK Shivaprakash SM Chakraborty SK Pharmacological studies on the venom of the marine snail Conus lentiginosus Reeve 1844. Int J Fish Aqua Stud 2014;1(3):79–85.

[17] Kaas Q Yu R Jin AH Dutertre S Craik DJ Conoserver: updated content, knowledge, and discovery tools in the conotoxin database. Nucleic Acids Res 2012;40(Database issue):D325–D330. 22058133 10.1093/nar/gkr886 PMC3245185

[18] Ellison J McIntosh M Olivera BM Alpha-conotoxin ІmЏ: similar α7 nicotinic receptor antagonists act at different sites. J Biol Chem 2003;278(2):757–764. 10.1074/jbc.M204565200 12384509

[19] Jimenez EC Olivera BM Divergent M-and O-super-family peptides from venom of fish-hunting Conus parius. Peptides 2010;31(9):1678–1683. 20570703 10.1016/j.peptides.2010.05.020 PMC2922443

[20] Sarasa A Mohammadi SA Christie MJ Conotoxin Interactions with α9α10-nAChRs: Is the α9α10-nicotinic acetylcholine receptor an important therapeutic target for pain management? Toxins 2015;7(10):3916–3932. 26426047 10.3390/toxins7103916 PMC4626711

[21] Moller C Vanderweit N Mari F Comparative analysis of proteases in the injected and dissected venom of cone snail species. Toxicon 2013;65:59–67. 23339854 10.1016/j.toxicon.2012.12.014 PMC3619401

[22] Violette A Biass D Dutertre S Koua D Piquemal D Pierrat F Large-scale discovery of conotoxins and conoproteins in the injectable venom of a fish-hunting cone snail using a combined proteomic and transcriptomic approach. J Proteom 2012;75(17):5215–5225. 10.1016/j.jprot.2012.06.001 22705119

[23] Khoobdel M Dehghani H Tavana AM Ghasemi M Dakhteh SM Hesni MA Rezaie-Atagholipour M Faunal data and envenomation emergency first aid of cone snails (Conus spp.) in Qeshm Island, the Persian Gulf. Asian Pac J Trop Med 2017;10(12):1167–1171. 29268973 10.1016/j.apjtm.2017.10.024

[24] Kruger NJ The Bradford method for protein quantitation. Methods Mol Biol 1994;32:9–15. 7951753 10.1385/0-89603-268-X:9

[25] Green BR Catlin P Zhang M Fiedler B Bayudan W Morrison A Conotoxins containing nonnatural backbone spacers: cladistic-based design, Chemical synthesis, and improved analgesic activity. J Biol Chem 2007;14(4):399–407. 10.1016/j.chembiol.2007.02.009 17462575

[26] Han TS Teichert RW Olivera BM Bulaj G Conus Venoms-A rich source of peptide-based therapeutics. Curr Pharm Des 2008;14(24):2462–2479. 18781995 10.2174/138161208785777469

[27] Lee S Kim Y Back SK Choi HW Lee JY Jung HH Analgesic effect of highly reversible ω- conotoxin FVIA on N-type Ca+2 channels. Mol Pain 2010;6:97–104. 21172037 10.1186/1744-8069-6-97 PMC3025903

[28] Shi G Liu Y Lin HM Yang SL Feng YL Reid PF Involvement of cholinergic system in suppression of formalin-induced inflammatory pain by cobratoxin. Acta Pharmacol Sin 2011;32(10):1233–1238. 21841815 10.1038/aps.2011.65 PMC4010082

[29] Barrot M Tests and models of nociception and pain in rodents. Neuroscience 2012;211:39–50. 22244975 10.1016/j.neuroscience.2011.12.041

[30] Tiwari G Tiwari R Sriwastawa B Bhati L Pandey S Pandey P Drug delivery systems: An updated review. Int J Pharm Invest 2012;2(1):2–11. 10.4103/2230-973X.96920 PMC3465154 23071954

[31] Zhang MM Green BR Catlin P Fiedler B Azam L Chadwick A Structure/function characterization of conotoxin KIIIA, an analgesic, nearly irreversible blocker of mammalian neuronal sodium channels. J Biol Chem 2007;282:30699–30706. 17724025 10.1074/jbc.M704616200

[32] Gatchel RJ Peng YB Peters ML Funchs PN Turk DC The biopsychosocial approach to chronic pain: scientific advances and future directions. Psychol Bull 2007;133(4):581–624. 17592957 10.1037/0033-2909.133.4.581

[33] Wu X Shao X Guo ZY Chi CW Identification of neuropeptide Y-like conotoxins from the venom of Conus betulinus. Acta Biochim Biophys Sin 2010;42(7):502–505. 20705590 10.1093/abbs/gmq042

[34] Elliger CA Richmond TA Lebaric ZN Pierce NT Sweedler JV Gilly WF Diversity of conotoxin types from Conus californicus reflects a diversity of prey types and a novel evolutionary history. Toxicon 2011;57(2):311–322. 21172372 10.1016/j.toxicon.2010.12.008 PMC3125295

[35] Olivera BM Teichert RW Diversity of the neurotoxic conus peptides. Mol Intervent 2007;7(5):251–260. 10.1124/mi.7.5.7 17932414

[36] Wen L Yang SH Zhou W Zhang Y Huang P New conotoxin so-3 targeting N-Type voltage-sensitive calcium channels. Mar Drugs 2006;4(3):215–227.

[37] Ochsner KN Ludlow DH Knierim K Hanelin J Ramachandran T Glover GC Neural correlates of individual differences in pain-related fear and anxiety. Pain 2006; 120(1–2):69–77. 16364548 10.1016/j.pain.2005.10.014 PMC2914607

[38] Kindler LL Bennett RM Jones KD Central sensitivity syndromes: mounting pathophysiologic evidence to link fibromyalgia with other common chronic pain disorders. Pain Manag Nurs 2011;12(1):15–24. 21349445 10.1016/j.pmn.2009.10.003 PMC3052797

[39] Zarrindast MR Pazouki M Nassiri-Rad S Involvement of cholinergic and opioid receptor mechanisms in nicotine-induced antinociception. Pharm Toxic 1997;81(5): 209–213. 10.1111/j.1600-0773.1997.tb00048.x 9396085

[40] McIntosh JM Corpuz GO Layer RT Garrett JE Wagstaff JD Bulaj G Isolation and characterization of a novel Conus peptide with apparent antinociceptive activity. J Biol Chem 2000;275(42):32391–32397. 10900201 10.1074/jbc.M003619200

[41] Bingham JP Baker MR Chun JB Analysis of a cone snail’s killer cocktail–The milked venom of Conus geographus. Toxicon 2012;60(6):1166–1170. 22884604 10.1016/j.toxicon.2012.07.014 PMC3696723

